# Genome-wide high-resolution mapping of DNA methylation identifies epigenetic variation across embryo and endosperm in Maize (*Zea may*)

**DOI:** 10.1186/s12864-014-1204-7

**Published:** 2015-01-23

**Authors:** Pengfei Wang, Han Xia, Ye Zhang, Shuzhen Zhao, Chuanzhi Zhao, Lei Hou, Changsheng Li, Aiqin Li, Chuanxi Ma, Xingjun Wang

**Affiliations:** Agricultural College, Anhui Agricultural University, Hefei, 230036 PR China; Bio-Tech Research Center, Shandong Academy of Agricultural Sciences; Shandong Provincial Key Laboratory of Crop Genetic Improvement, Ecology and Physiology, Jinan, 250100 PR China

**Keywords:** DNA methylation, Maize, Embryo, Endosperm, Transposable element, Imprinting gene, MeDIP-seq

## Abstract

**Background:**

Epigenetic modifications play important roles in plant and animal development. DNA methylation impacts the transposable element (TE) silencing, gene imprinting and expression regulation.

**Results:**

Through a genome-wide analysis, DNA methylation peaks were characterized and mapped in maize embryo and endosperm genome, respectively. Distinct methylation level was observed across maize embryo and endosperm. The maize embryo genome contained more DNA methylation than endosperm. Totally, 985,478 CG islands (CGIs) were identified and most of them were unmethylated. More CGI shores were methylated than CGIs in maize suggested that DNA methylation level was not positively correlated with CpG density. The promoter sequence and transcriptional termination region (TTR) were more methylated than the gene body (intron and exon) region based on peak number and methylated depth. Result showed that 99% TEs were methylated in maize embryo, but a large portion of them (34.8%) were not methylated in endosperm. Maize embryo and endosperm exhibit distinct pattern/level of methylation. The most differentially methylated region between embryo and endosperm are CGI shores. Our results indicated that DNA methylation is associated with both gene silencing and gene activation in maize. Many genes involved in embryogenesis and seed development were found differentially methylated in embryo and endosperm. We found 41.5% imprinting genes were similarly methylated and 58.5% imprinting genes were differentially methylated between embryo and endosperm. Methylation level was associated with allelic silencing of only a small number of imprinting genes. The expression of maize DEMETER-like (DME-like) gene and MBD101 gene (MBD4 homolog) were higher in endosperm than in embryo. These two genes may be associated with distinct methylation levels across maize embryo and endosperm.

**Conclusions:**

Through MeDIP-seq we systematically analyzed the methylomes of maize embryo and endosperm and results indicated that the global methylation status of embryo was more than that of the endosperm. Differences could be observed at the total number of methylation peaks, DMRs and specific methylated genes which were tightly associated with development of embryo and endosperm. Our results also revealed that many DNA methylation regions didn’t affect transcription of the corresponding genes.

**Electronic supplementary material:**

The online version of this article (doi:10.1186/s12864-014-1204-7) contains supplementary material, which is available to authorized users.

## Background

DNA methylation, a conserved epigenetic mechanism involved in many important biological processes, is associated with gene silencing, X chromosome inactivation in females, and maintenance of genomic integrity in eukaryotes [1-3]. DNA methylation protects against transposon proliferation and impacts genomic imprinting [4-6].

Similar to mammalian genomes, DNA methylation in plant genomes predominantly occurs at CpG site. This is maintained by METHYLTRANSFERASE1 (MET1), a homolog of DNA methyltransferase1 (Dnmt1). In addition, plants DNA methylation occurs at CpHpG and CpHpH sites, and is maintained by CHROMOMETHYLASE3 (CMT3) [7-9]. In plant, DNA glycosidase subfamily including DEMETER (DME) and Repressor of Silencing 1 (ROS1) could mediate demethylation [10,11]. Although the methylated cytosine contexts in animals and plants are different, DNA methylation is conserved in both TEs and genes. In animals, plants and fungi, the active genes are generally unmethylated, while TEs are heavily methylated. However, green algae have an unusual pattern of methylation compared to other eukaryotes. More methylation was detected in exons to compare with TEs regions [12-14].

There is strong evidence that DNA methylation in promoter region represses gene expression [15-20]. In rice, methylation of transcriptional termination region (TTR) showed stronger repression effect on gene expression to compare with promoter methylation [21]. Results showed that gene-body methylation were positively associated with gene expression [22-24]. However, DNA methylation in the first exon was found to associate with gene silencing [25,26].

Bisulfite sequencing has been used to detect the methylated cytosines [27]. This method is very accurate to find the methylated cytosine of the individual locus. However, it is difficult to explore genome-wide methylation. MSAP (methylation sensitive amplification polymorphism) has been used to explore the genome-wide methylation, but it can only detect few methylation fractions and is limited by the types of enzymes used. Sequencing-based and microarray-based high-throughput detection of DNA methylation approaches are widely used in genome-wide methylation studies. DNA methylation microarray, DNA immunoprecipitation combined with high-throughput sequencing (MeDIP-seq) and bisulfite libraries construction combined with high-throughput sequencing are proved to be efficient [4,21,28-30]. These approaches have been used to discover global methylation dynamics in different plant species including *Arabidopsis* [4,7,30,31], sorghum [32] and rice [21,33].

Several studies reported the differential DNA methylated regions which were correlated with variable gene expression within the examined tissues [21,34]. Results showed that the difference of DNA methylation could only account for a limited extend of gene expression variation among plant vegetative tissues [21,35].

DNA methylation is very important for plant embryogenesis and seed development. Abnormal embryo methylation causes defect in embryogenesis, such as abnormal of cell division, embryo apical domain aberrance and reduction of viability [8]. In seed plants, gene imprinting occurs in endosperm [31,36-38]. Studies showed that the expression of only a small portion of imprinting genes was correlated with DNA methylation in *Arabidopsis* [31,39,40]. The expression level of maize imprinting genes was much higher in endosperm than in embryo [39,41]. TEs exhibited toxic effects on genome, and embryo represses parasitic TEs to prevent damage of genome during seed development. DNA methylation on TEs is an important way to repress TEs [42]. TEs silencing relies mainly on RNAi pathway directed methylation, and siRNA is the major mediator for CpHpH DNA methylation [22,43]. Large amount of small RNA was accumulated in rice endosperm. However, the CpHpH methylation level of endosperm is quite low, while the embryo CpHpH methylation level is rather high. The major methylation in rice endosperm is CpHpH, suggesting RNAi pathway does not participate in endosperm DNA methylation. The endosperm derived small RNAs could be transported to embryo where they mediate TEs silencing by DNA methylation [7,31,33,44].

In plants, endosperm DNA was hypomethylated in various sequence contexts. For example, rice endosperm CG methylation is about 93% of the methylation rate in embryos. CHG and CHH methylation is by 2–5 folds lower in endosperm compared to embryo. In *Arabidopsis,* CG methylation of most loci is demethylated in endosperm [12]. In *Arabidopsis* ecotype Col-gl and Ler, thousands of genes exhibited higher level of methylation in embryo than that in endosperm [31].

Previous studies on DNA methylation in embryo and endosperm using DNA methylation microarray, immunoprecipitation and MSAP have assessed only a small portion of tissue-specific DNA methylation variations in maize genome [29,31,35,37,45,46]. In the current study, we used Solexa MeDIP-seq to profile the methylomic landscape across embryo and endosperm, comparing the differences of their methylation modes. Many genes that were differentially methylated between embryo and endosperm were identified.

## Results

### Methylomic profiling of embryo and endosperm in maize

**Figure 1:**
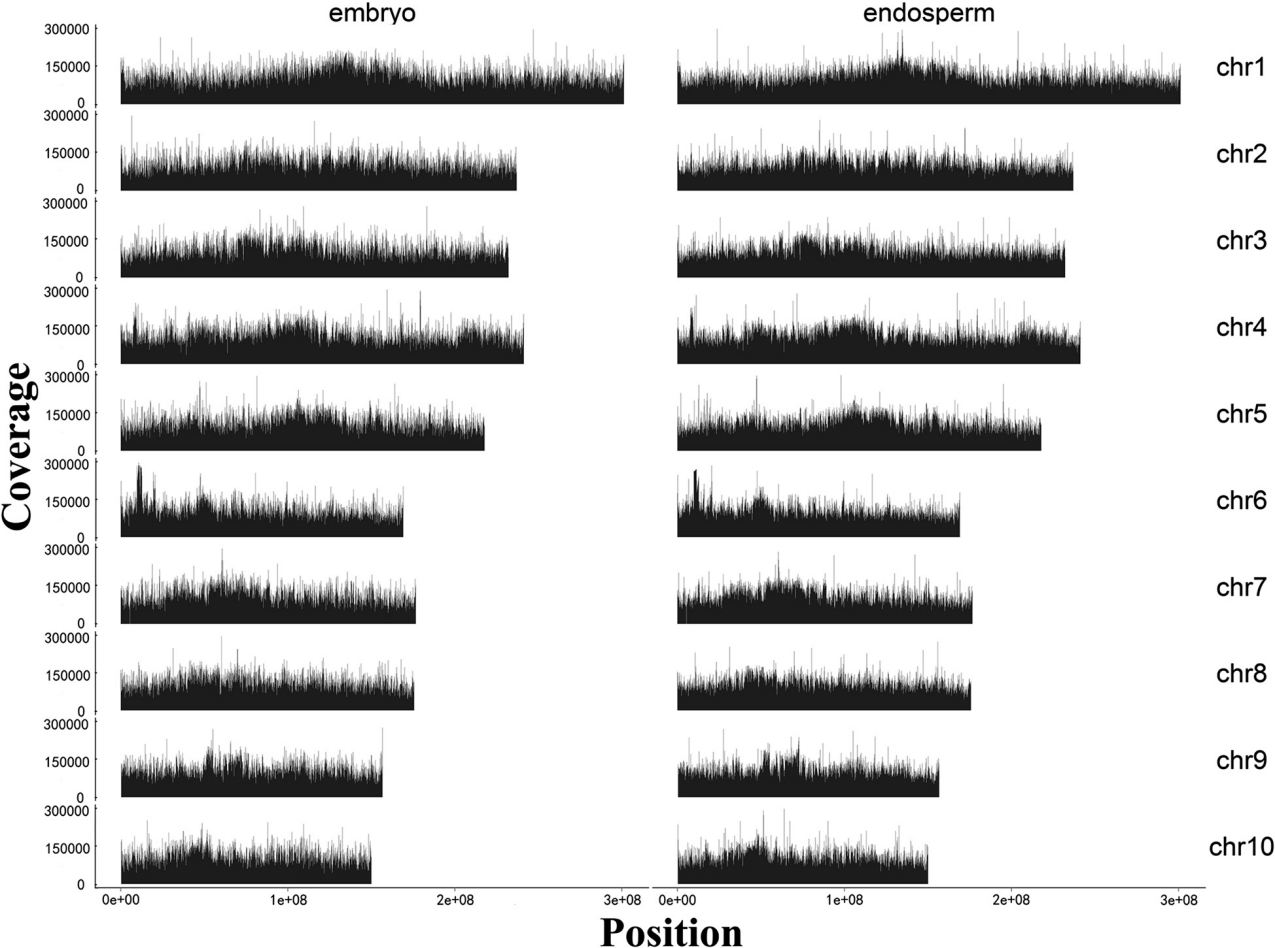
**Chromosomal distribution of DNA methylation read of maize endosperm and embryo.** Each chromosome was split in 10 k wide windows and the methylated read count was calculated for each window in embryo and endosperm. Y-axis is the read count mapped in each window.

We generated a total of 2,748,497,900 bp of DNA immunoprecipitation sequencing (MeDIP-seq) data from maize endosperm and 2,807,090,100 bp data from maize embryo. From endosperm and embryo, 53,541,909 and 54,639,671 clean reads (average length 50 bp) were obtained, respectively. More than 96% of MeDIP-seq reads were aligned (mapped) on maize genome in each tissue (Table 1). Figure 1 showed the chromosomal distribution of DNA methylation read of maize endosperm and embryo.

**Table 1:** **Solexa MeDIP-seq data**

**Sample**	**Total reads**	**Clean reads**	**Mapped reads**	**Mapping ratio**
Endosperm	54,969,958	53,541,909	51,643,126	(96.45%)
Embryo	56,141,802	54,639,671	52,648,132	(96.36%)

**Figure 2:**
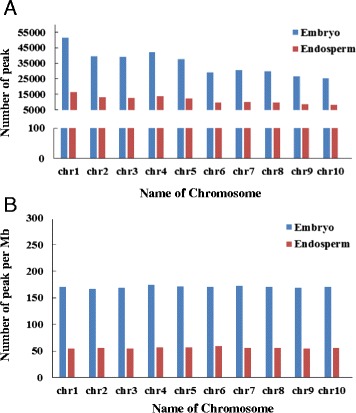
**Distribution of DNA methylation peaks in chromosomes. A**, Distribution of DNA methylation peaks in maize embryo and endosperm chromosomes. **B**, The number of methylation peaks per Mb in maize embryo and endosperm chromosomes.

The mapped reads were used in scanning the methylation peak with Model-based analysis of chIP-seq (MACS) (version1.4). A total number of 115,599 methylation peaks (diffScore ≥ 50, p ≤ 1e-5, diffScore = −10*LOG10pvalue) from endosperm and 353,232 methylation peaks from embryo were identified (Figure 2). DNA methylation peak number shows the popularity of methylation in genome. More DNA methylation peaks mean more loci in genome are methylated.

### Characterization of methylated DNA regions

We analyzed the methylation status of CpG islands (CGIs), CGI shores (spanning 2,000 bp up-and down-stream of each CGI) as well as other locations in the genome. CpG island in maize genome was identified using CpG report software (EMBOSS: 6.4.0.0). The default parameters are as following: the minimum length is 200 bp, minimum observed/expected value is 0.6, the minimum percentage of CpG content is 50% (http://emboss.bioinformatics.nl/cgi-bin/emboss/newcpgreport). In total, 985,478 CGIs were identified in this study. Additional file 1: File S1a, Additional file 2: File S1b, Additional file 3: File S1c lists all CpG islands of whole maize genome. The locations of CpG islands on chromosomes, the length of the island and the observed/expected value were also provided. 2000 bp up- or down-stream of CpG island was considered to be the CpG shore [28]. If a CpG shore was overlapped with the methylation region identified in this study, we considered that this CpG shore was methylated.

**Figure 3:**
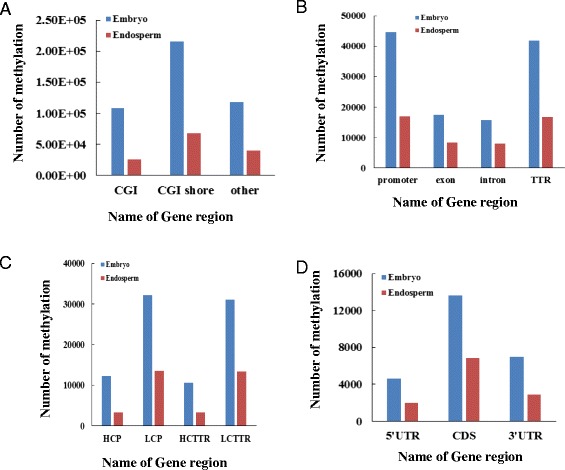
**Distribution of DNA methylated peaks in gene regions.** Distribution of DNA methylation peaks in CGIs, CGI shores and other regions **(A)**, Distribution of DNA methylation peaks in TTRs, promoters, exons and introns **(B)**, Distribution of DNA methylation peaks in HCP, LCP, HCTTR and LCTTR **(C)**. Distribution of DNA methylation peaks in CDs, 5’UTR and 3’UTR **(D)**.

There were 108,441 methylated CGIs and 214,787 methylated CGI shores in embryo, and 26,009 methylated CGIs and 67,483 methylated CGI shores in endosperm. In both embryo and endosperm, less CGIs were methylated compared to CGI shores (Figure 3A). Most CG islands were unmethylated (only 11% of CGIs in embryo and 2.6% CGIs in endosperm were methylated), which was in agreement with the methylation profiles in human and pig [28,47,48]. Methylation status of embryo and endosperm was analyzed in promoter, exon, intron, transcriptional termination region, 5’ UTR, 3’ UTR and coding sequences (Additional file 4: Table S1a, Additional file 5: Table S1b). Gene regions that overlapped with DNA methylation peaks were considered methylated. Methylated promoters and transcriptional termination region (TTR) could be classified into four types by CpG content: high CpG content promoters (HCPs), low CpG content promoters (LCPs), high CpG content TTRs (HCTTRs) and low CpG content TTRs (LCTTRs) as previously described [28,48,49].

We detected 16,835 methylated promoters and 16,758 methylated TTRs in maize endosperm genome, while 44,488 methylated promoters and 41,715 methylated TTRs in maize embryo were identified. The DNA methylation occurred more frequently in promoter and TTR regions than other gene regions (Figure 3B).

**Figure 4:**
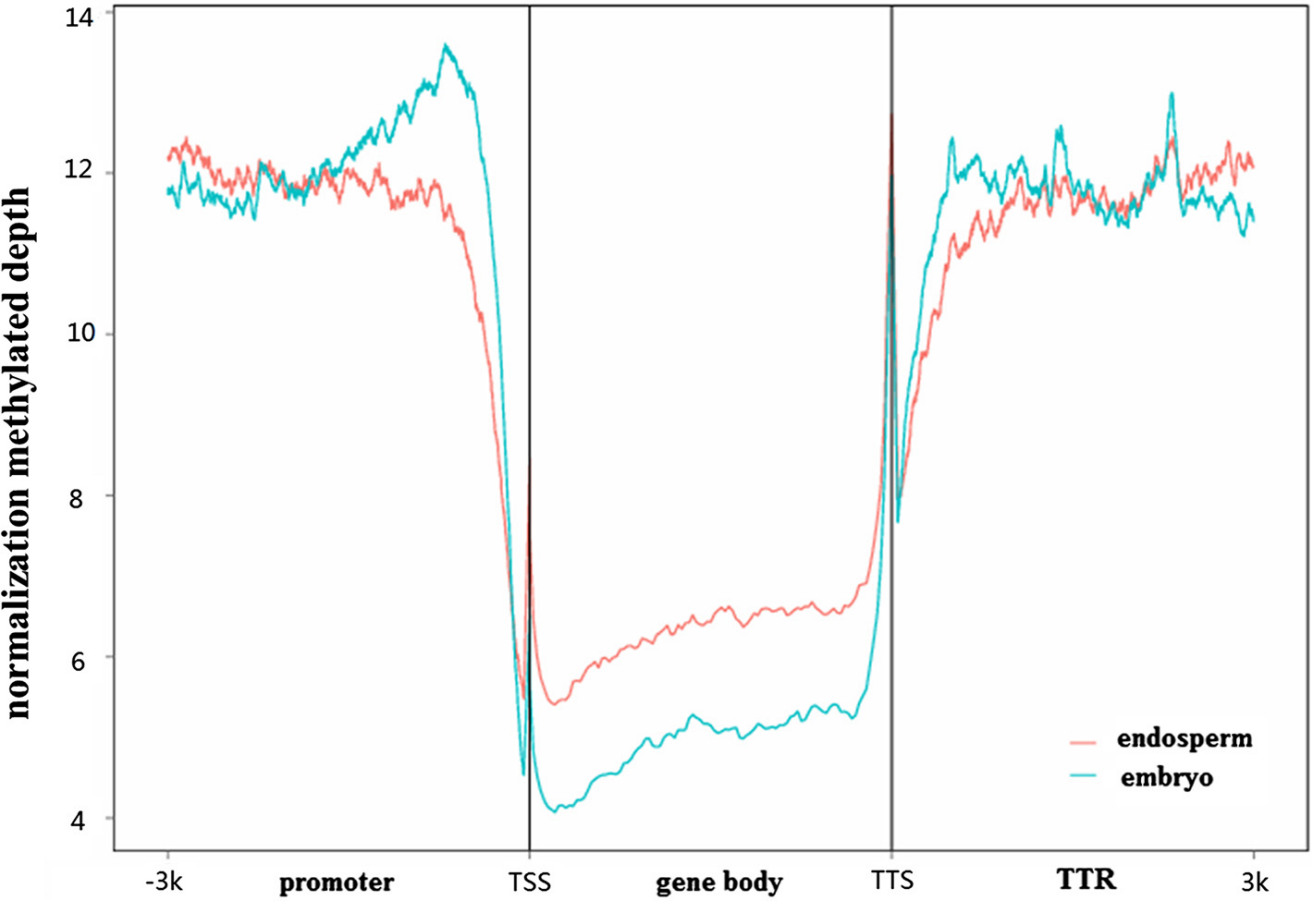
**DNA methylation level in gene body, promoter and transcription termination region.**

In embryo, 12,313 methylated HCPs, 42,790 methylated LCPs, 10,628 methylated HCTTRs and 39,982 methylated LCTTRs were detected. Endosperm contained much less methylated HCPs (3,284), LCPs (16,835), HCTTRs (3,351) and LCTTRs (16,757) (Figure 3C). We also analyzed the methylation levels within introns and exons in embryo and endosperm. There were 17,475 methylated exons and 15,703 methylated introns in embryo, and 8,388 methylated exons, 7,970 methylated introns in endosperm. Figure 4 shows that the promoter and TTR were more methylated than the gene body (intron and exon) on average.

The transposase genes that overlapped with DNA methylation peak were considered methylated TEs. In maize seed, we detected 184 methylated TEs which contain MuDR transposon, gypsy-type retrotransposon, copia sub-class retrotransposon, CACTA sub-class transposon and other types of transposons by BLASTX against nr protein database. Only two of these TEs were not methylated in embryo, while 64 of them were not methylated in endosperm (Additional file 6: Table S2).

### Characterization of Differential Methylated Region (DMR)

We identified DNA methylation regions using a newly developed method. Any peak detected in embryo overlapping with peaks in endosperm, we will select the genomic region covering them as one DNA methylation region. If a peak detected in embryo (or endosperm) doesn’t overlap with any peak from endosperm (or embryo), we also considered the genomic region covering the peak to be a DNA methylation region in embryo or endosperm. Thus, 381,221 DNA methylation regions were identified in this study.

The read number of each methylated region from embryo or endosperm was used to calculate the normalized log_2_ value (log_2_ ratio of read number of embryo versus endosperm) and test p-value using the DEGseq R package. If normalized log_2_ value >0 (or read number from endosperm in the methylation region =0) and p < 0.001, the methylated region was considered up-methylated in embryo, and down-methylated in endosperm. If normalized log_2_ value < 0(or read number from embryo in the methylation region =0) and p < 0.001, the methylated region was considered up-methylated in endosperm, and down-methylated in embryo.

Among the identified 381,221 methylated regions, 238,088 regions were differentially methylated between embryo and endosperm (Additional file 7: Table S3). Totally, 175,337 and 62,751 differentially methylated regions (DMRs) were up-methylated in embryo and endosperm, respectively.

**Figure 5:**
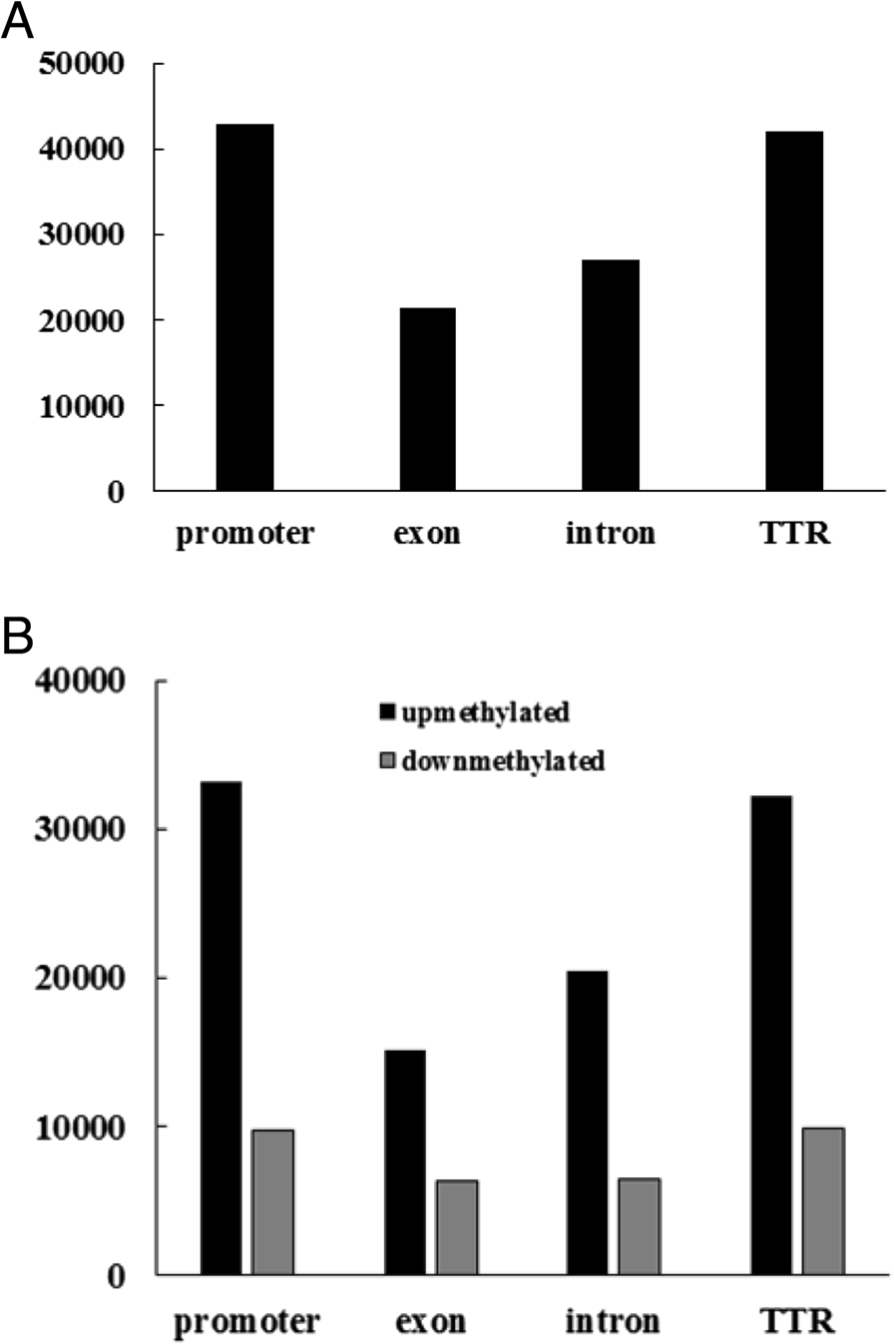
**Distribution of DMRs in maize gene regions. A**, Distribution of DMRs in TTRs, promoters, exons and introns. **B**, Distribution of up-methylated and down-methylated DMRs in embryo TTRs, promoters, exons and introns.

**Figure 6:**
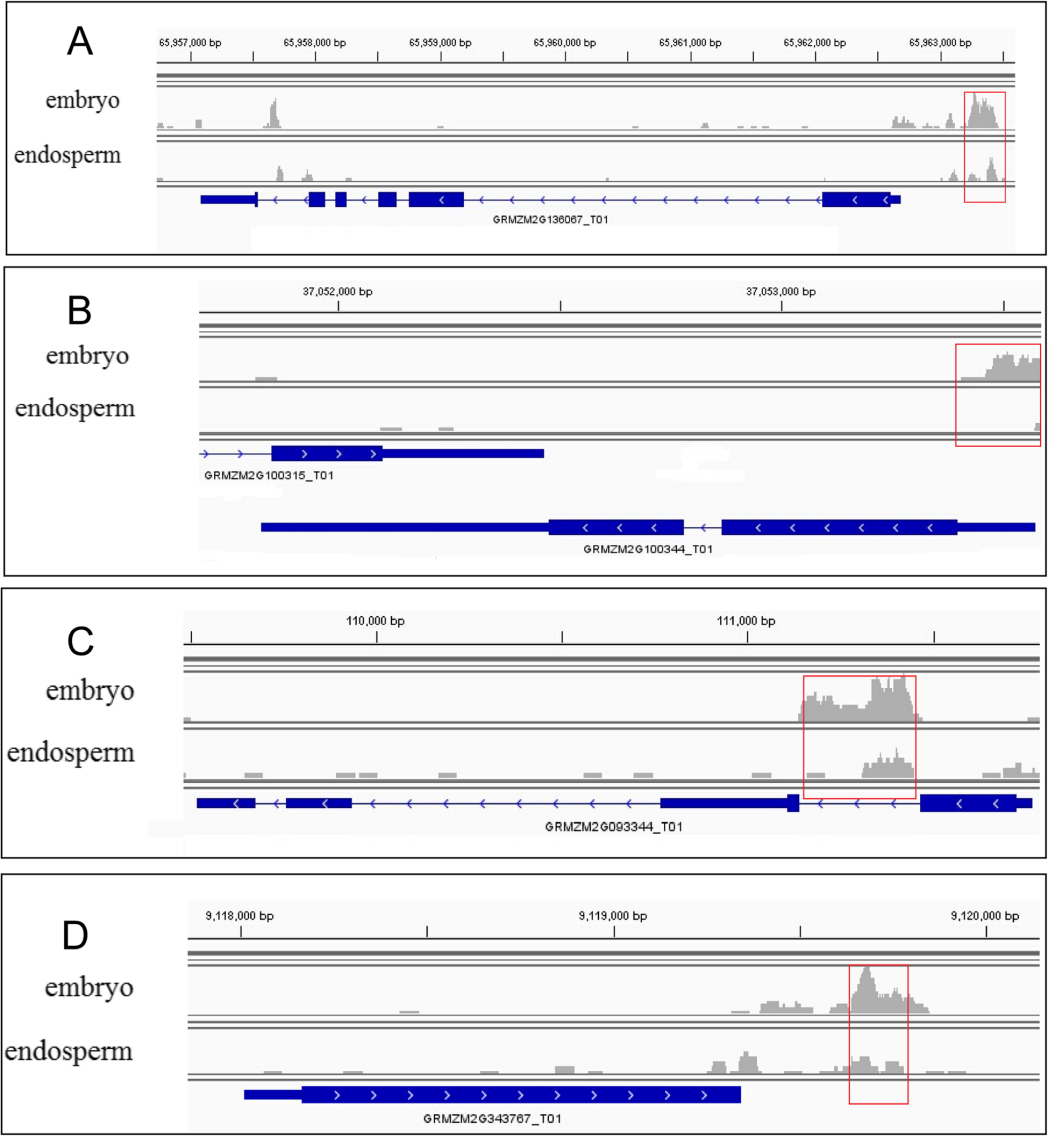
**Promoter, TTR, intron and exon overlapped up-methylated DMRs in embryo.** Some gene regions overlapped with the up-methylated DMRs in embryo, such as GRMZM2G136067 promoter **(A)**, GRMZM2G343767 TTR **(B)**, GRMZM2G100344 exon **(C)** and GRMZM2G093344 intron **(D)**. Red boxes were the DMRs that were up-methylated in embryo and down-methylated in endosperm. The arrow on gene structure represents the direction of transcription.

More DMRs were located in CGI shore to compare with CGI. In promoter and TTR, we found more DMRs to compare with intron and exon. In embryo, more up-methylated DMRs were distributed in promoters and TTRs than intron and exon, while more down-methylated DMRs were distributed in promoter and TTR in endosperm. In embryo, more up-methylated DMRs could overlap with promoter, TTR, intron and exon to compare with endosperm (Figure 5). The overlapped up-methylated DMRs of some promoters, TTRs, introns and exons were plotted using the Integrative Genomics Viewer (IGV) software (Figure 6).

### Function annotation of the methylated genes

Function annotation of the methylated genes was carried out by BLASTX against non-redundant (nr) protein database. Information from proteins with the highest similarity to the given methylated gene was used to annotate the gene function. The encoding proteins of the methylated genes were further compared with KEGG (Kyoto Encyclopedia of Genes and Genomes). Totally, 15,958 methylated genes were annotated by BLASTX analysis. Some ribosomal protein, storage protein, LEA (late embryogenesis abundant) protein encoding genes and imprinting genes were found methylated. Many genes encoding transcription factors, such as WRKY, SBP, NAC, MYB, bZIP families were methylated (Additional file 8: Table S4).

The methylated genes were predicted to be involved in 118 metabolic pathways based on KEGG database. Many methylated genes encoding proteins involved in chromatin structure and DNA synthesis, cell cycle regulation, nitrogen metabolism, fatty acid synthesis and elongation, starch and sugar metabolism, amino acid metabolism, protein metabolism, brassinosteriod biosynthesis, tricarboxylic acid cycle pathway, hormone metabolism and signal transduction pathways (Additional file 9: Figure S1-9). These results indicated that DNA methylation was involved in a wide range of biological processes.

### Differentially methylated genes in maize embryo and endosperm

Of the 15,958 annotated methylated genes, 296 in embryo, and 7,735 in endosperm were de-methylated. Many genes were only methylated in embryo but not in endosperm and many genes are more methylated in embryo than endosperm (all DMRs that the gene contained is up-regulated in embryo than in endosperm). Some of these genes may contribute to epigenetic inheritance and reprogramming across generations, for example, gene encoding DNA-3-methyladenine glycosylase I, gene encoding histone deacetylase, lysine-specific histone demethylase and histone-arginine methyltransferase. Some genes are associated with cell differentiation and vascular development, for example, the ZF-HD-type transcription factor, TCP-1/cpn60 chaperonin family protein and TCP family protein. Some genes are involved in hormone metabolism and signal transduction pathways, such as ABI3/VP1 type transcription factor, auxin response factor (ARF), Aux/IAA family, GRAS family transcription factor, B3 DNA binding domain family protein, GID1-like gibberellin receptor, and BRASSINOSTEROID INSENSITIVE 1-associated receptor kinase 1. Gene involved in cell fate determination, GeBP type transcription factor [50], was identified. WOX family proteins, key regulators of embryo development, were detected in this study. Genes encoding key enzymes in starch synthesis, for example, starch synthase I, starch branching enzyme IIb and granule-bound starch synthase precursor were detected differentially methylated. Dicer-like (DCL) and Argonaute (AGO), key enzymes of small RNA biogenesis pathway were found to be more methylated in embryo than in endosperm. bHLH and MADS-box transcription factor also showed more methylation in embryo than in endosperm (Additional file 10: Table S5).

### Gene Ontology (GO) enrichment of differentially methylated genes

All methylated genes were annotated based on GO annotation. Additional file 11: Table S6 shows the GO categories and function of methylated genes.

To better understand the potential function of the differential methylated genes, GO functional classification of these genes was performed by Blast2GO program. Fisher’s exact test p-values were calculated for overrepresentation of the differential methylated genes (genes contained DMRs) in all GO categories. GO terms with p < 0.05 were considered as significant enriched. Totally, 97 GO terms were significantly enriched, with 37 in cellular component, 19 in molecular function and 41 in biological process. In biological process, the most significantly enriched differentially methylated genes are related to photosynthesis, electron transport chain, and respiratory electron transport chain. In cellular component, the most significantly enriched genes are related to chloroplast, photosynthetic membrane, and thylakoid part. While, in molecular function, the most significantly enriched genes are involved in quinone binding, NADH dehydrogenase (quinone) and NADH dehydrogenase (ubiquinone) activity (Table 2).

**Table 2:** **GO function analysis of the enriched differential methylated genes**

**Category**	**GO term**	**Count**	**P-value**
**biological process**			
**GO:0015979**	photosynthesis	224	9.30E-13
**GO:0022900**	electron transport chain	232	2.13E-11
**GO:0022904**	respiratory electron transport chain	98	5.01E-10
**GO:0006091**	generation of precursor metabolites and energy	384	9.60E-10
**GO:0006119**	oxidative phosphorylation	89	1.29E-08
**GO:0042773**	ATP synthesis coupled electron transport	89	1.29E-08
**GO:0015980**	energy derivation by oxidation of organic compounds	142	1.39E-07
**GO:0045333**	cellular respiration	135	1.67E-07
**GO:0019684**	photosynthesis, light reaction	140	2.64E-07
**GO:0009767**	photosynthetic electron transport chain	59	2.87E-07
**GO:0009772**	photosynthetic electron transport in photosystem II	25	2.79E-06
**GO:0044237**	cellular metabolic process	3685	5.18E-06
**GO:0009058**	biosynthetic process	1708	7.56E-06
**GO:0044249**	cellular biosynthetic process	1597	3.61E-05
**GO:0009987**	cellular process	4664	0.00011
**GO:0015985**	energy coupled proton transport, down electrochemical gradient	46	0.00014
**GO:0015986**	ATP synthesis coupled proton transport	46	0.00014
**GO:0042777**	plasma membrane ATP synthesis coupled proton transport	18	0.00016
**GO:0006351**	transcription, DNA-dependent	206	0.00017
**GO:0032774**	RNA biosynthetic process	207	0.00032
**GO:0009145**	purine nucleoside triphosphate biosynthetic process	69	0.00033
**GO:0009206**	purine ribonucleoside triphosphate biosynthetic process	69	0.00033
**GO:0034645**	cellular macromolecule biosynthetic process	940	0.00035
**GO:0009059**	macromolecule biosynthetic process	947	0.00093
**GO:0009201**	ribonucleoside triphosphate biosynthetic process	70	0.00185
**GO:0009142**	nucleoside triphosphate biosynthetic process	72	0.00189
**GO:0015672**	monovalent inorganic cation transport	142	0.00216
**GO:0009152**	purine ribonucleotide biosynthetic process	81	0.00222
**GO:0006818**	hydrogen transport	77	0.00226
**GO:0015992**	proton transport	77	0.00226
**GO:0042775**	mitochondrial ATP synthesis coupled electron transport	56	0.00272
**GO:0006754**	ATP biosynthetic process	63	0.00586
**GO:0006164**	purine nucleotide biosynthetic process	84	0.01066
**GO:0006120**	mitochondrial electron transport, NADH to ubiquinone	38	0.01086
**GO:0010467**	gene expression	846	0.01137
**GO:0072522**	purine-containing compound biosynthetic process	88	0.01641
**GO:0008152**	metabolic process	4538	0.01879
**cellular component**			
**GO:0009507**	chloroplast	1513	2.21E-14
**GO:0034357**	photosynthetic membrane	277	9.70E-14
**GO:0044436**	thylakoid part	289	1.13E-13
**GO:0042651**	thylakoid membrane	268	1.87E-13
**GO:0055035**	plastid thylakoid membrane	263	2.47E-13
**GO:0009535**	chloroplast thylakoid membrane	262	3.82E-13
**GO:0009534**	chloroplast thylakoid	290	7.41E-13
**GO:0031976**	plastid thylakoid	290	7.41E-13
**GO:0009579**	thylakoid	345	4.30E-12
**GO:0031984**	organelle subcompartment	294	4.46E-12
**GO:0009539**	photosystem II reaction center	20	2.60E-08
**GO:0009536**	plastid	2256	7.71E-07
**GO:0009523**	photosystem II	62	1.57E-06
**GO:0009521**	photosystem	74	1.78E-06
**GO:0030076**	light-harvesting complex	22	9.18E-06
**GO:0044422**	organelle part	1690	1.14E-05
**GO:0044446**	intracellular organelle part	1686	1.25E-05
**GO:0033177**	proton-transporting two-sector ATPase complex, proton-transporting domain	34	1.40E-05
**GO:0032991**	macromolecular complex	1156	2.06E-05
**GO:0045263**	proton-transporting ATP synthase complex, coupling factor F(o)	27	2.74E-05
**GO:0005761**	mitochondrial ribosome	41	3.68E-05
**GO:0045259**	proton-transporting ATP synthase complex	43	5.44E-05
**GO:0016469**	proton-transporting two-sector ATPase complex	57	6.01E-05
**GO:0005840**	ribosome	337	0.00012
**GO:0044434**	chloroplast part	663	0.00018
**GO:0044429**	mitochondrial part	220	0.00049
**GO:0044391**	ribosomal subunit	179	0.00068
**GO:0044435**	plastid part	671	0.00069
**GO:0005762**	mitochondrial large ribosomal subunit	14	0.00184
**GO:0005739**	mitochondrion	1597	0.00213
**GO:0043234**	protein complex	769	0.00294
**GO:0000313**	organellar ribosome	41	0.00436
**GO:0005759**	mitochondrial matrix	70	0.00484
**GO:0015935**	small ribosomal subunit	88	0.01036
**GO:0030075**	plasma membrane-derived thylakoid	9	0.01078
**GO:0030096**	plasma membrane-derived thylakoid photosystem II	9	0.01078
**GO:0030529**	ribonucleoprotein complex	394	0.01593
**GO:0005753**	mitochondrial proton-transporting ATP synthase complex	29	0.01639
**GO:0016021**	integral to membrane	882	0.03048
**GO:0044425**	membrane part	1118	0.03143
**GO:0044444**	cytoplasmic part	5004	0.03237
**molecular function**			
**GO:0048038**	quinone binding	69	1.29E-10
**GO:0050136**	NADH dehydrogenase (quinone) activity	86	2.42E-10
**GO:0008137**	NADH dehydrogenase (ubiquinone) activity	82	2.07E-09
**GO:0003954**	NADH dehydrogenase activity	86	6.51E-09
**GO:0019843**	rRNA binding	92	3.35E-08
**GO:0003899**	DNA-directed RNA polymerase activity	93	4.58E-08
**GO:0034062**	RNA polymerase activity	95	8.32E-08
**GO:0016655**	oxidoreductase activity, acting on NADH or NADPH, quinone or similar compound as acceptor	91	5.39E-07
**GO:0016651**	oxidoreductase activity, acting on NADH or NADPH	117	5.94E-06
**GO:0045156**	electron transporter, transferring electrons within the cyclic electron transport pathway of photosynthesis activity	25	2.61E-05
**GO:0005198**	structural molecule activity	325	0.00023
**GO:0046933**	hydrogen ion transporting ATP synthase activity, rotational mechanism	34	0.00055
**GO:0003735**	structural constituent of ribosome	254	0.00058
**GO:0032549**	ribonucleoside binding	31	0.00065
**GO:0001882**	nucleoside binding	31	0.00135
**GO:0015078**	hydrogen ion transmembrane transporter activity	99	0.00302
**GO:0015077**	monovalent inorganic cation transmembrane transporter activity	134	0.00421
**GO:0016779**	nucleotidyltransferase activity	200	0.00936
**GO:0016984**	ribulose-bisphosphate carboxylase activity	18	0.04269

### Methylation and transcriptional repression of imprinting genes

Previous studies demonstrated that some imprinting genes in endosperm were associated with DNA methylation [31,39-41]. In maize endosperm, 17.3% of the analyzed imprinting genes showed differential methylation between the two parental alleles [39,41]. We examined the correlation between expression of imprinting genes and DNA methylation level in embryo and endosperm. We analyzed the methylation mode of 176 imprinting genes identified by Zhang [39]. Only 65 out of the 176 imprinting genes were detected to be methylated in embryo or endosperm. Thirty eight of the methylated genes were only methylated in embryo, suggesting that these imprinting genes were not associated with allelic silencing in endosperm. Five of the methylated genes were only methylated in endosperm, suggesting that these imprinting genes were not associated with allelic silencing in embryo.

Based on the transcription data [37], we found 25 of the methylated genes showed much higher expression level in endosperm than in embryo (endosperm/embryo average RPKM >1.5), among them, the majority (17/25) of genes were more methylated or only methylated in embryo, four of them were more methylated in endosperm or only methylated in endosperm and the methylation level of rest four genes was similar between embryo and endosperm.

15 of the methylated genes showed much higher expression level in embryo than in endosperm (embryo/endosperm average RPKM >1.5), among them, one of the 15 genes was more methylated in endosperm, 6 were more methylated in embryo, and the rest 8 genes showed similar methylation level between embryo and endosperm.

The rest 25 methylated genes showed similar expression level between endosperm and embryo, among them, ten of the 25 genes were more methylated in embryo, the rest 15 genes showed similar methylation level between embryo and endosperm. Totally, among the 65 imprinting genes which were methylated in embryo or endosperm, 41.5% were methylated similarly between embryo and endosperm, while 58.5% were differentially methylated (Additional file 12: Table S7). These results showed that only a small portion of the imprinting genes were regulated by DNA methylation.

**Figure 7:**
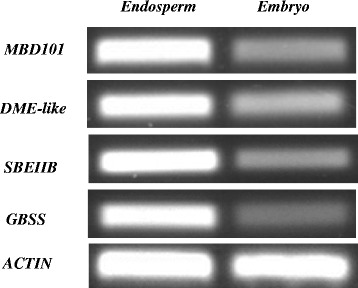
**Different expression of DME-like gene, MBD4-like gene, SBEIIB gene and GBSS gene in maize embryo and endosperm.**

A maize DME-like gene (GRMZM2G123587) and a MBD4-like gene (GRMZM5G847045) were identified in this study. The DME-like gene encodes 5-methylcytosine DNA glycosylase, and MBD4-like gene encodes methylation-binding domain 101 protein. Maize DME-like (GenBank: AFW71475.1) is homologous to Arabidopsis DME (AAM77215.1, identifies = 64%, E-value = 0) and contains HhH-GPD base excision DNA repair protein domain. Maize MBD4-like NP_001105172.1 is homologous to Arabidopsis MBD4 (NP_191862.1, identifies = 47%, E-value = 2e-37) and contains methyl-CpG-binding domain. DME participates in demethylation of the maternal genome in endosperm [10,11]. Overexpression of MBD4/AID gene caused bulk genome de-methylation in zebra fish [9]. The expression levels of DME-like and MBD4-like gene were all higher in maize endosperm (Figure 7). Therefore, high transcriptional activity of these two genes could be associated with the low methylation level in endosperm.

## Discussion

Our data showed the different pattern of DNA methylation between maize embryo and endosperm. Embryo contained more DNA methylation peaks to compare with endosperm in each chromosome.

More DNA methylation peaks were located in CGI shores compared to CGIs and other gene regions, which is in agreement with the results from human. CGI shore also contained more DMRs than CGI. More DMRs were distributed in promoters and TTRs than introns and exons. Many studies showed that LCPs were more methylated than HCPs [49], we found the same result in maize. In maize, we found that TTRs methylation may have similar function to promoter methylation, which is in agreement with previous study [21]. We found that LCTTRs contained more DNA methylation peaks than the HCTTRs. More DMRs were up-methylated in embryo than in endosperm. This methylation may cause tissue-specific expression of genes between embryo and endosperm. Our results showed that 58.5% imprinting genes were differentially methylated between embryo and endosperm. Methylation level could be negatively or positively correlated with the expression level of imprinting genes. Methylation level was associated with allelic silencing of only a small number of imprinting genes.

Starch is a major component of maize endosperm and comprises two different forms of carbohydrate polymers: a linear amylose and a branched amylopectin. Our results showed that the genes encoding starch synthase I, starch branching enzyme IIb (SbeIIb) and granule-bound starch synthase precursor (GBSS) were more methylated in embryo than in endosperm. The expression levels of these genes were much higher in endosperm than in embryo, which was consistent with previous transcriptome data [37]. Both GBSS and starch synthase I showed embryo specific DNA methylated, and the methylated regions were located in promoter. SbeIIb gene promoter was methylated only in embryo and intron was more methylated in embryo than in endosperm. This is in agreement with previous studies that SbeII and GBSS promoters are endosperm-specific promoters [51,52]. Tissue-specific promoters may contain tissue-specific cis-elements, for example, RY motif [51,52] or other elements, for example, GGATCC palindrome, which could be recognized by DNA methyltransferases [53]. GGATCC was detected in maize SbeIIb gene promoter. It is possible that the promoters of the above described three genes were de-methylated in endosperm, and promoter methylation of these genes in embryo was associated with their transcription repression. In addition, genes in starch synthesis and metabolism, such as isoamylase-type starch debranching enzyme ISO3, starch branching enzyme IIa, starch synthase IIIb-1, starch phosphorylase and starch binding domain containing family protein genes were all methylated mainly in promoter region only in embryo. These genes were hypermethylated in embryo and hypomethylated in endosperm.

Storage protein zein and oleosin genes were found to be more methylated in embryo than in endosperm. Zein gene expression level was higher in endosperm than in embryo [37]. The 15 kDa beta zein, 22 kDa alpha zein1, 22 kDa alpha zein 4 and 22 kDa alpha zein 5 encoding genes were methylated specifically in embryo. The DMR of 15 kDa beta zein encoding gene was located in TTR, while the DMRs of other three genes all located in exon. The methylated level and the expression level of zeins were negatively correlated. Oleosins are key components of oil body. In maize seed, the expression level of 16 kDa oleosin gene and Zm-II oleosin gene was higher in embryo compared to endosperm. However, our data clearly showed that these two genes were methylated in embryo but not in endosperm. The methylation was located in promoter and TTR regions. The methylation level of these oleosin genes was not negatively correlated with their expression.

WUSCHEL-related homeobox (WOX) transcription factor is necessary for cell division that forms the apical domain of embryo. In maize embryo, the WOX8 promoter and TTR was hypermethylated, however, this gene was highly expressed in embryo. In embryos of *Arabidopsis* met1-6 mutant, expression level of de-methylated WOX8 is lower than the hypermethylated WOX8 in wild-type embryo [8]. This result suggested that methylation could enhance WOX8 expression in embryo. Gene methylation could repress or active gene expression, and sometimes gene methylation may not correlate with transcription [21].

A hypothesis is that TE silencing is through the RNAi pathway. Small RNAs may be transported from endosperm to embryo where they lead to siRNA or miRNA-mediated methylation of TEs. The evidence is that abundant TE-derived small RNAs were accumulated in endosperm in *Arabidopsis*, but extremely low levels of CHH methylation occurs in endosperm. In contrast, high CHH methylation was detected in embryo [54]. In other words, the link between RNAi and DNA methylation may be weakened in endosperm and the small RNAs could be taken away. In our study, TEs were more methylated in embryo than in endosperm, possibly due to the imported small RNA. If this is true, it could explain, at least in part, the hypomethylation of endosperm. We identified a maize DME-like gene and a MBD4-like gene, and found that their expression levels were higher in maize endosperm than in embryo. DME and MBD4 could mediate strong bulk genome DNA demethylation. Therefore, the differentially expressed DME-like and MBD101 genes could be a possible reason for the differential methylation patterns in embryo and endosperm.

## Conclusions

Through MeDIP-seq we systematically analyzed the methylomes of maize embryo and endosperm and results indicated that the global methylation status of embryo was more than that of the endosperm. Differences could be observed at the total number of methylation peaks, DMRs and specific methylated genes which were tightly associated with development of embryo and endosperm. Our results also revealed that many DNA methylation regions didn’t affect transcription of the corresponding genes.

## Methods

### Tissue collection and genomic DNA extraction

Maize endosperm and embryos were collected from B73 ears 14 days after self-pollination (DAP). The genomic DNA of two tissues was extracted by CTAB method. Tissues were ground with liquid nitrogen and 0.1 g powder was transferred into 2.0 ml tube containing 600 μl 2% CTAB solution (65°C) and 1 μl 10 μM RNase. Sample was incubated in 65°C water bath for 20 min, then mixed with 300 μl chloroform and 300 μl Tris saturated Phenol. The sample was centrifuged at 12000 rpm for 10 min at 4°C. Supernatant was transferred into a new tube and mixed with 600 μl chloroform, centrifuged at 12000 rpm for 10 min at 4°C. Supernatant was mixed with 2 volume of 100% ethanol for DNA precipitation. After centrifugation, the liquid was discarded and the precipitated DNA was washed with 70% ethanol. Dried the DNA briefly and then dissolved in 30 μl double distilled water.

### MeDIP libraries construction and sequencing

DNA was sheared using the Bioruptor sonicator (Diagenode). End reparation, base addition and adaptor ligation were performed using Methyl-Seq 1 Kit. Adaptor-ligated sheared DNA was immunoprecipitated by 5-methylcytidine antibody with Magnetic Methylated DNA Immunoprecipitation kit (Diagenode) to construct the MeDIP libraries. Each MeDIP library was subjected to high-throughput sequencing by Illummina solexa HiSeq2000 platform.

### Sequencing quality control and reads processing

Sequencing data was analyzed by data collection software. Quality control was performed using FastQC (http://www.bioinformatics.babraham.ac.uk/projects/fastqc/). Clean reads were generated by using fastx software (version: 0.0.13) (http://hannonlab.cshl.edu/fastx_toolkit/index.html).

Genome mapping was performed using Bowtie2 (version: 2.0.5) software with default parameters [55] to generate the BAM files. The plant chloroplast genome has no methylation activity [56], so we only accounted the nuclear methylation reads detected in genome.

The peaks were detected from the BAM files using MACS (version: 1.4) [57]. Peaks with p-value = < 1e-5 were selected for further analysis.

The MeDIP-seq data from this study have been submitted to the NCBI Gene Expression Omnibus (http://www.ncbi.nlm.nih.gov/geo) under accession no. GSE58549 (http://www.ncbi.nlm.nih.gov/geo/query/acc.cgi?acc=GSE58549).

### Classfication of methylated genomic regions

Based on maize genome database transcript annotation and our methylation data, we detected the methylated (overlapped regions with DNA methylation peaks) exon, intron, promoter, CD, 3’ UTRs, 5’UTR and TTR in maize. Methylated promoters and TTRs were classified into four types: high CpG content promoters (HCPs), low CpG content promoters (LCPs), high CpG content TTRs (HCTTRs) and low CpG content TTRs (LCTTRs) according to CpG content as previously described [49].

### CGIs and CGI shores identification

CGIs and CGI shores (2000 bp up- and down-stream of CGI) of maize genome were predicted by new CpG report software (Version: EMBOSS: 6.4.0.0) (http://emboss.bioinformatics.nl/cgi-bin/emboss/newcpgreport). The default parameters are as following: the minimum length is 200 bp, minimum observed/expected value is 0.6, the minimum percentage of CpG content is 50%.

### Identification of DME-like gene and a MBD101 gene

We employed the protein sequences of Arabidopsis DME gene (AT5G04560.1) and MBD4 gene (AT3G63030.1) as queries to identify the most possible maize DME and MBD4 gene in genome database using BLASTp program (E-value <10). Sequences with the smallest E-value and the highest identities were considered DME and MBD4 homologs. A search on the Pfam database was performed to confirm the sequences.

### RT-PCR analyses

Total RNA was prepared using Trizol agent (TaKaRa, Dalian, China) according to the manufacturer’s instructions. For reverse transcription, after DNase I treatment, the first-strand cDNA was synthesized with an oligo (dT) primer using a PrimeScript™ first-strand cDNA synthesis kit (D6110A; TaKaRa, Dalian, China). Equal amounts of RT products were used to perform subsequent PCR amplification. Primers used to amplify DME-like were 5’-CACAAACCCAGGAAACGGAG-3’ and 5’-ACCACCCCAACCCCAATG-3’. Primers used to amplify MBD4-like were 5’-AACATACCAAAGCCTCCACCA-3’ and 5-TGCCTCCAGAAACTTATCCACA -3’. Primers used to amplify the control, Actin 1, were 5’-GGGATTGCCGATC GTATGAG-3’ and 5’-GAGCCACCGATCCAGACACT-3’. Primers used to amplify SBEIIB were 5’-ACACCGGCCTCTTCTTAACTC-3’ and 5’-CTCGCCCTCAGGAACCAT-3’. Primers used to amplify GBSS were 5’-CTGAGCCTCAACAACAACCC-3’ and 5’-TGTAGATGCCGTGGGACTG-3’.

## Additional files

Additional file 1: File S1a. CGI location of maize genome. Additional file 2: File S1b. CGI location of maize genome. Additional file 3: File S1c. CGI location of maize genome. Additional file 4: Table S1a. Methylated gene regions in maize embryo. The first three rows represent the position of DNA methylation peaks in the genome, the fourth row is the length of the peaks, the fifth row is the number of read in each peak, the sixth row is the diffScore of the peaks, and the seventh row is gene regions that overlapped with the DNA methylation peaks. Additional file 5: Table S1b. Methylated gene regions in endosperm. The first three rows represent the position of DNA methylation peaks in the genome, the fourth row is the length of the peaks, the fifth row is the number of read in each peak, the sixth row is the diffScore of the peaks, and the seventh row is gene regions that overlapped with the DNA methylation peaks. Additional file 6: Table S2. Methylated transposable elements in maize embryo and endosperm. The first row is the gene ID contains TE, in the second row “ + ” represents that TE is methylated in the embryo, “-” represents that TE is not methylated in the embryo, in the third row “ + ” represents that TE is methylated in the endosperm, and “ - ” represents that the TE is not methylated in the endosperm. Additional file 7: Table S3. DMRs in maize endosperm and embryo. The first three rows of the table represent the position of DMR, the fourth row is the length of the DMR, the fifth and sixth rows represent the number of read of each DMR in the embryo and endosperm, the seventh row represents the log_2_ of DMR, The eighth row is normalized log_2_, the ninth row is exact test p-value. Additional file 8: Table S4. Nr annotation of methylated genes in maize embryo and endosperm. Additional file 9: Figure S1. Methylated genes involved in TCA cycle. Genes in the red boxes were methylated. **Figure S2.** Methylated genes involved in fatty acid biosynthesis. Genes in the red boxes were methylated. **Figure S3.** Methylated genes involved in fatty acid metabolism. Genes in the red boxes were methylated. **Figure S4.** Methylated genes involved in starch and sucrose metabolism. Genes in the red boxes were methylated. **Figure S5.** Methylated genes involved in ribosome biosynthesis. Genes in the red boxes were methylated. **Figure S6.** Methylated genes involved in RNA polymerase. Genes in the red boxes were methylated. **Figure S7.** Methylated genes involved in basal transcription factors. Genes in the red boxes were methylated. **Figure S8.** Methylated genes involved in DNA replication. Genes in the red boxes were methylated. **Figure S9.** Methylated genes involved in plant hormone signal transduction. Genes in the red boxes were methylated. Additional file 10: Table S5. GO annotation of maize methylated genes in embryo and endosperm. Additional file 11: Table S6. Differentially methylated genes in maize embryo and endosperm. Gene region of each gene in the endosperm or embryo, “ + ” represents methylation, “-” represents does not methylated. Additional file 12: Table S7. Methylation status and expression level of imprinting genes. Each gene region of imprinted genes in the embryo or endosperm, “ + ” represents methylation, “-” represents does not methylated. RPKM represents the expression of the gene in Embryo or endosperm.
