# Supplementary material for: Genome-wide high-resolution mapping of DNA methylation identifies epigenetic variation across embryo and endosperm in Maize (Zea may)
Source: BMC Genomics. 2015 Jan 23;16(1):21. doi: 10.1186/s12864-014-1204-7 (PMC4316406; doi:10.1186/s12864-014-1204-7)
Supplement: Additional file 9: Figure S1. — Methylated genes involved in TCA cycle. Genes in the red boxes were methylated. Figure S2. Methylated genes involved in fatty acid biosynthesis. Genes in the red boxes were methylated. Figure S3. Methylated genes involved in fatty acid metabolism. Genes in the red boxes were methylated. Figure S4. Methylated genes involved in starch and sucrose metabolism. Genes in the red boxes were methylated. Figure S5. Methylated genes involved in ribosome biosynthesis. Genes in the red boxes were methylated. Figure S6. Methylated genes involved in RNA polymerase. Genes in the red boxes were methylated. Figure S7. Methylated genes involved in basal transcription factors. Genes in the red boxes were methylated. Figure S8. Methylated genes involved in DNA replication. Genes in the red boxes were methylated. Figure S9. Methylated genes involved in plant hormone signal transduction. Genes in the red boxes were methylated. [file 12864_2014_1204_MOESM9_ESM.ppt]

## Slide 1
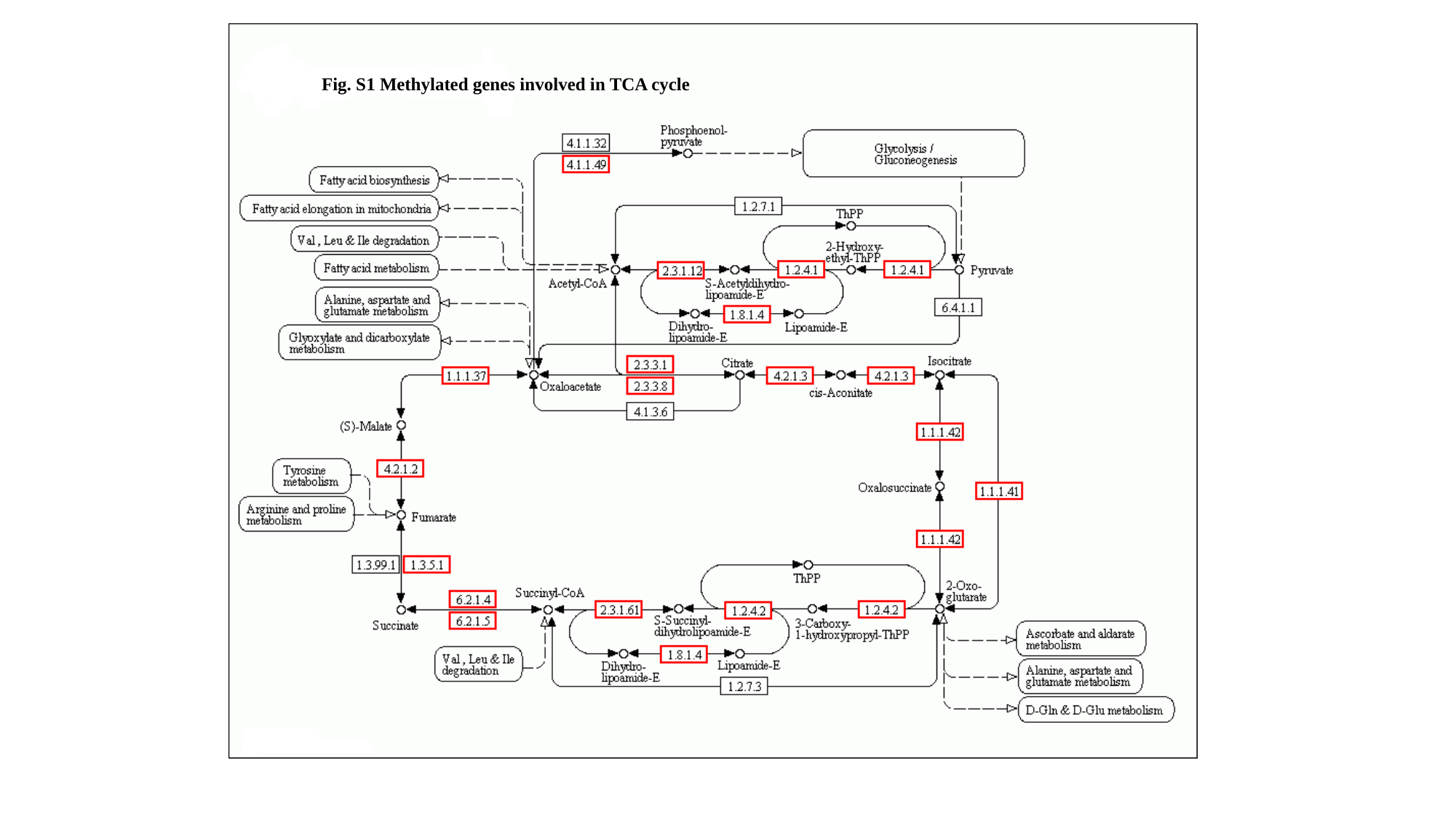

Fig. S1 Methylated genes involved in TCA cycle

## Slide 2
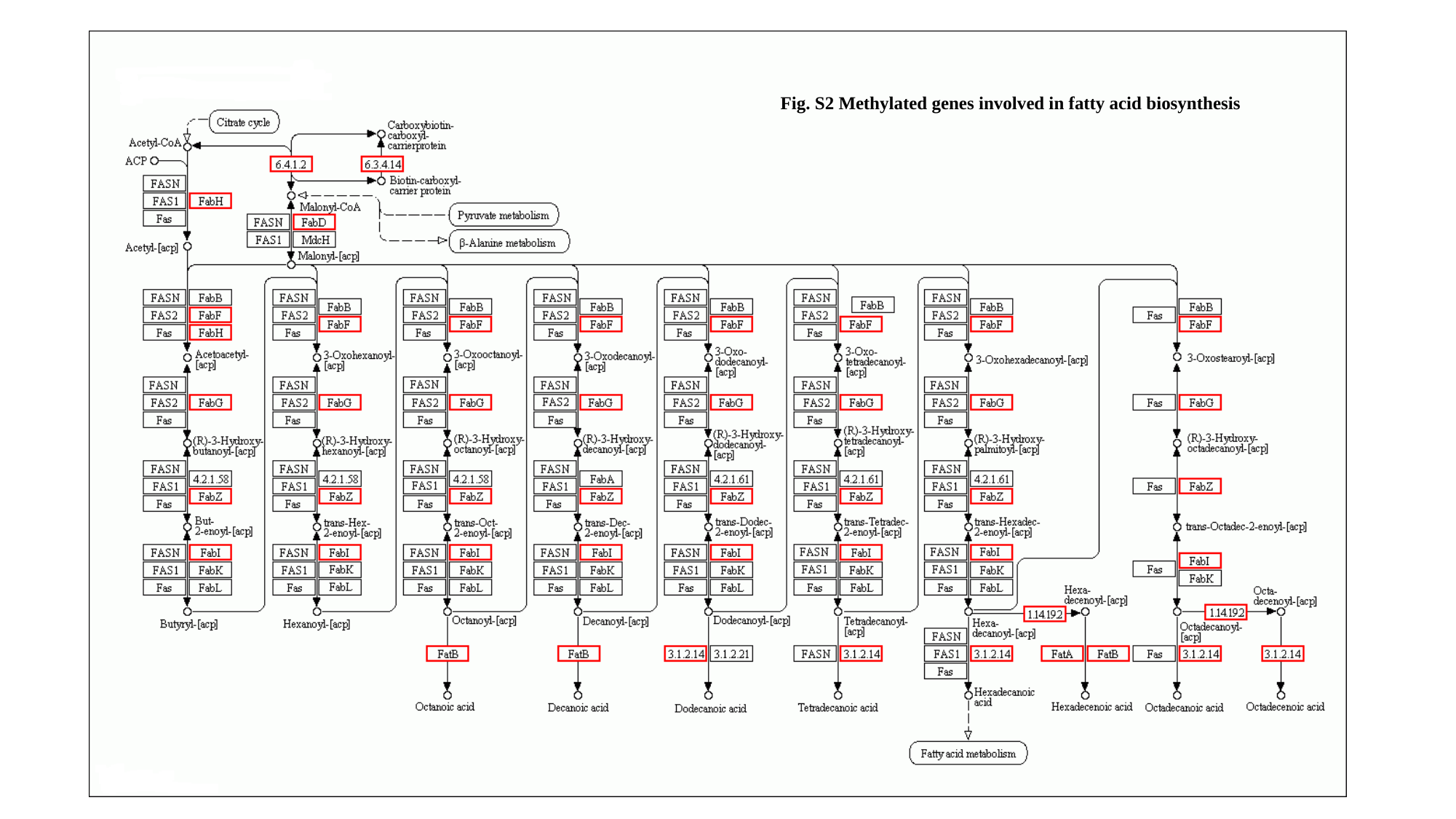

Fig. S2 Methylated genes involved in fatty acid biosynthesis

## Slide 3
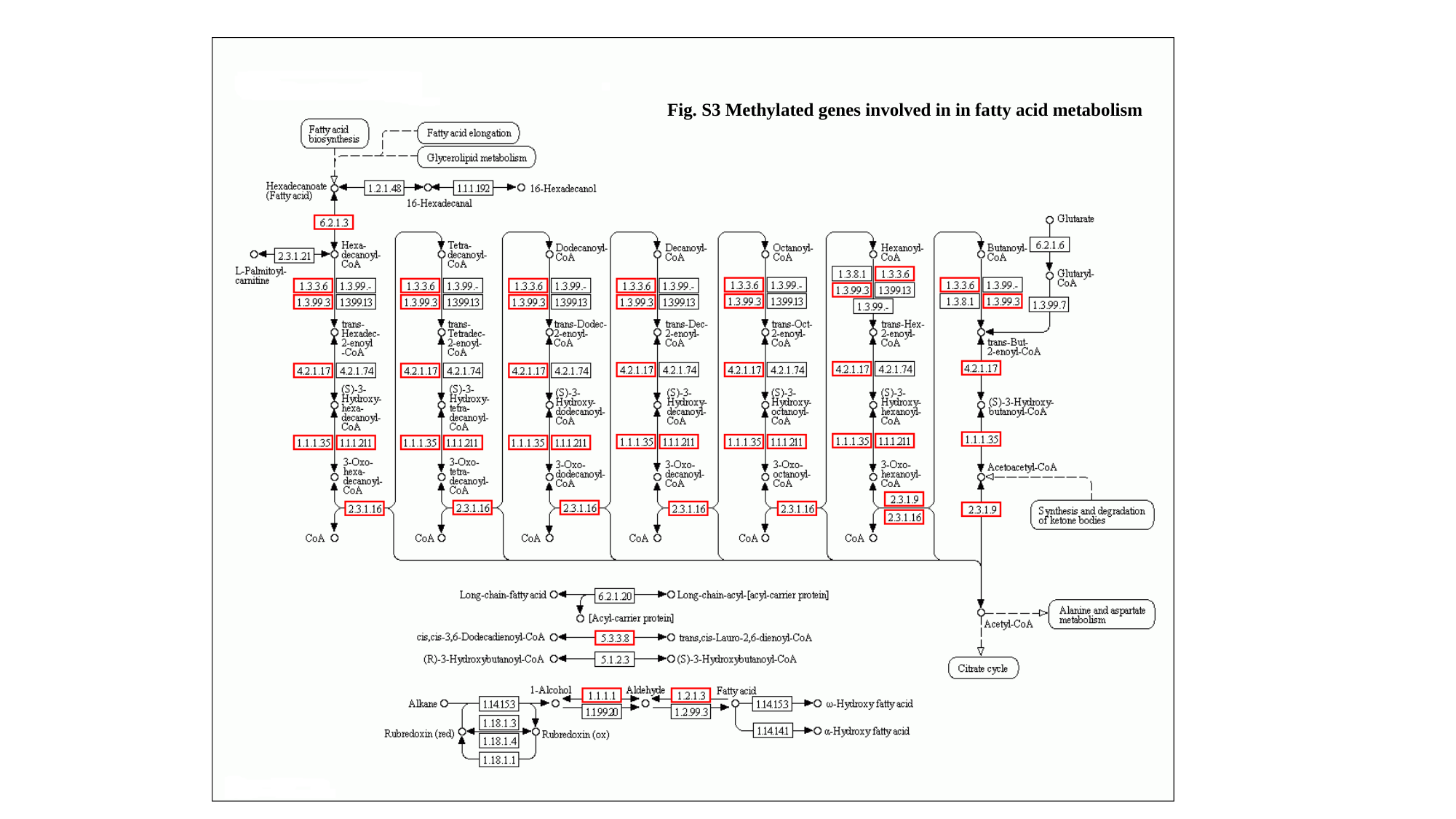

Fig. S3 Methylated genes involved in in fatty acid metabolism

## Slide 4
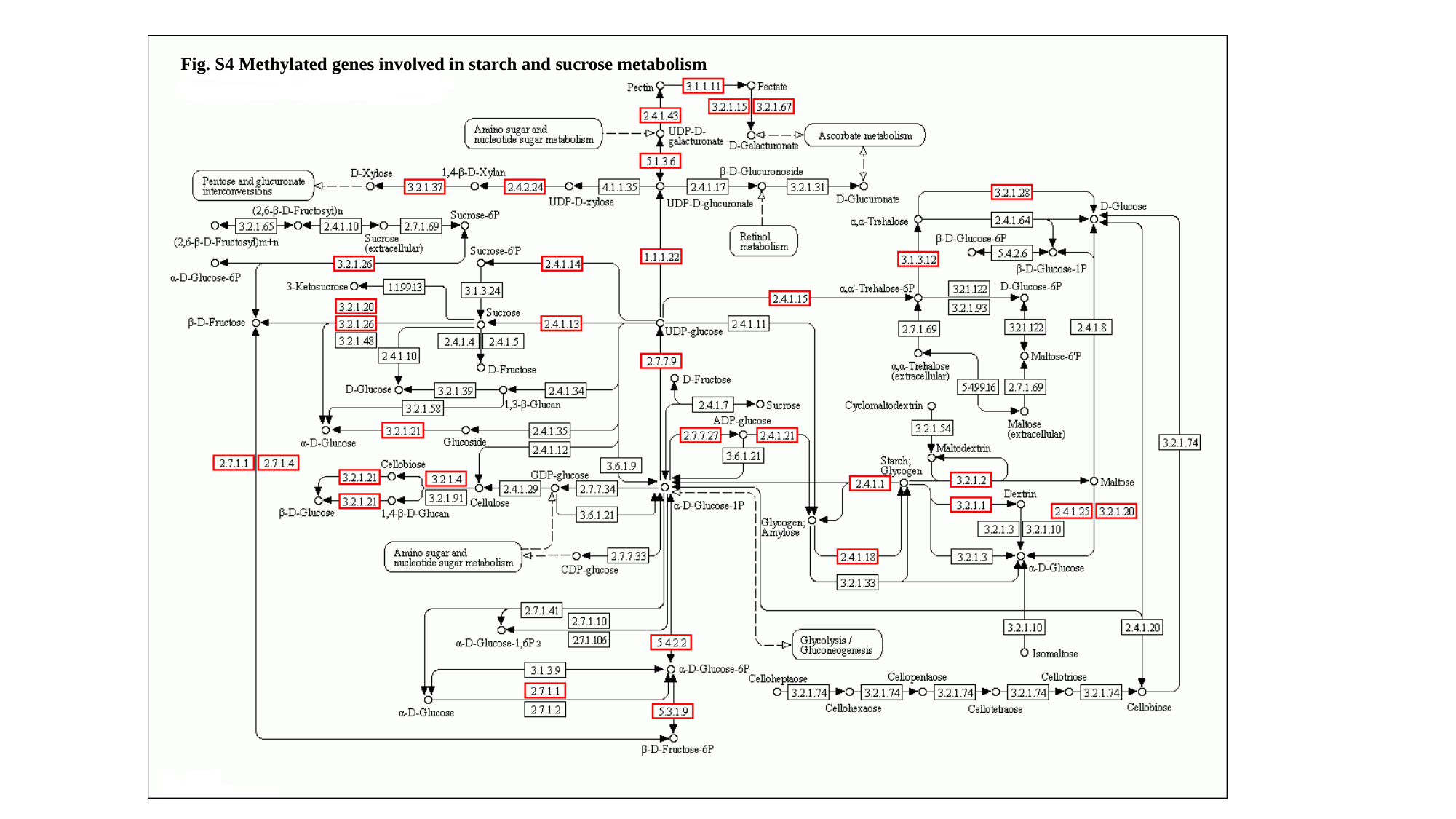

Fig. S4 Methylated genes involved in starch and sucrose metabolism

## Slide 5
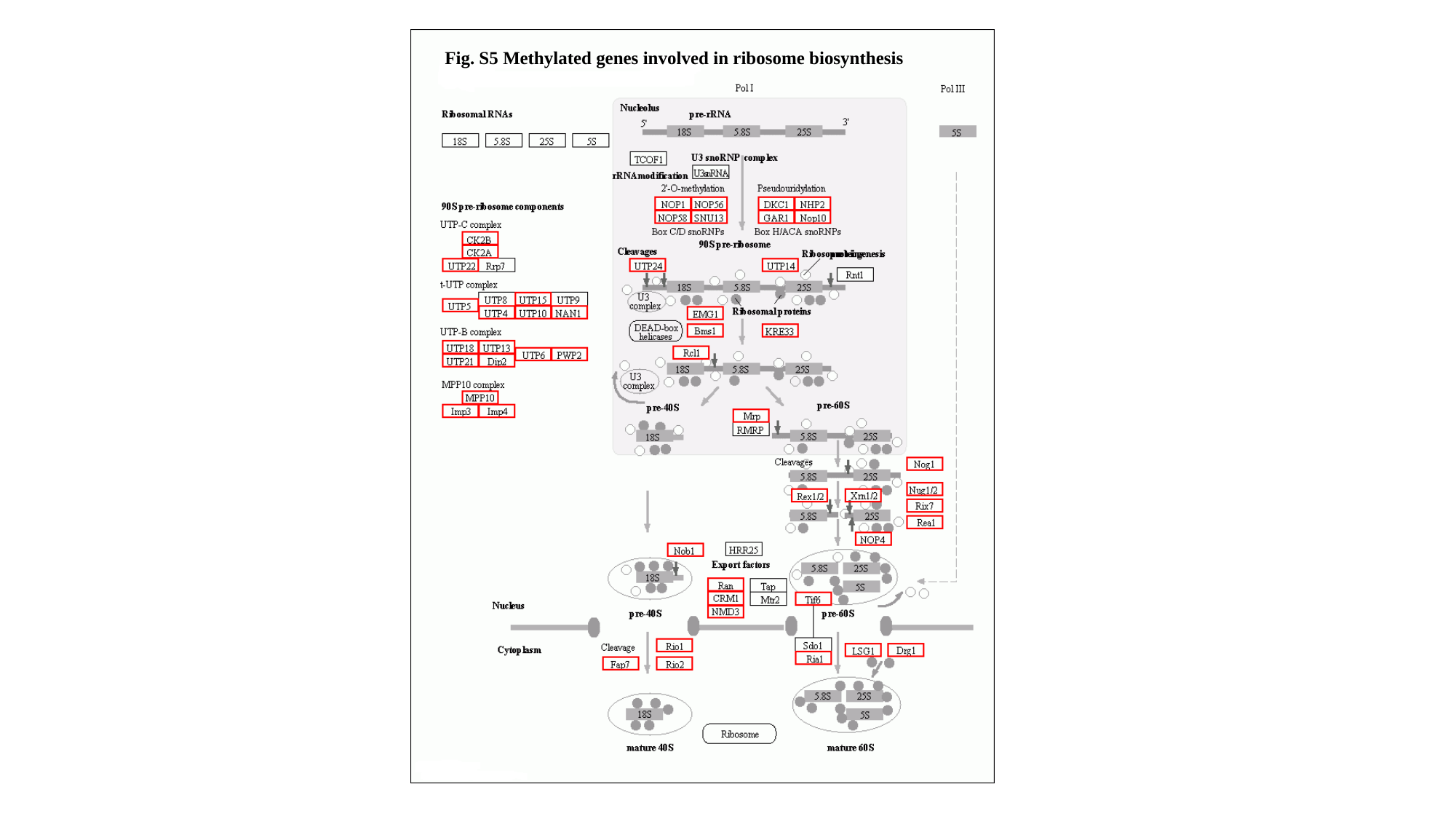

Fig. S5 Methylated genes involved in ribosome biosynthesis

## Slide 6
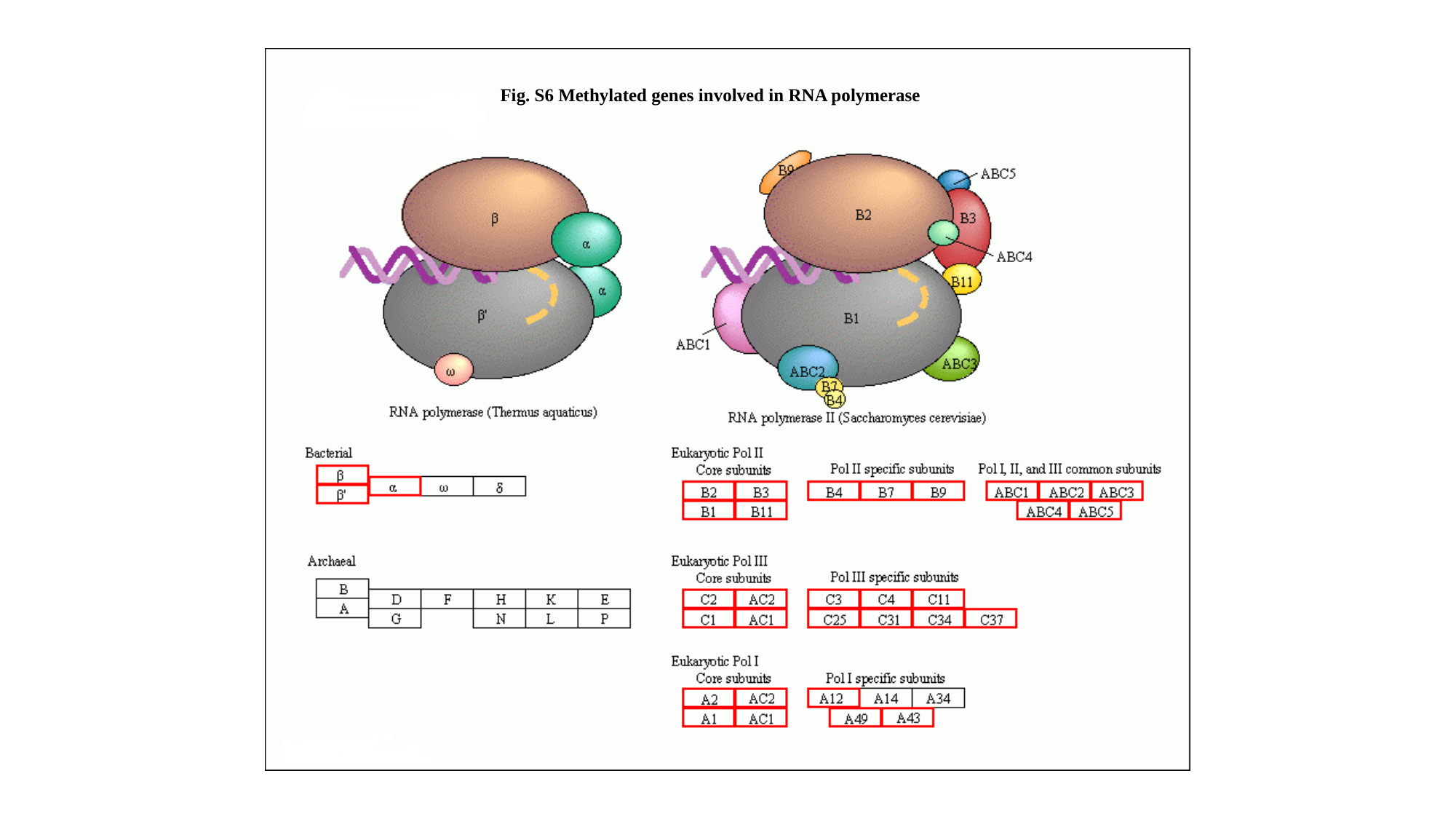

Fig. S6 Methylated genes involved in RNA polymerase

## Slide 7
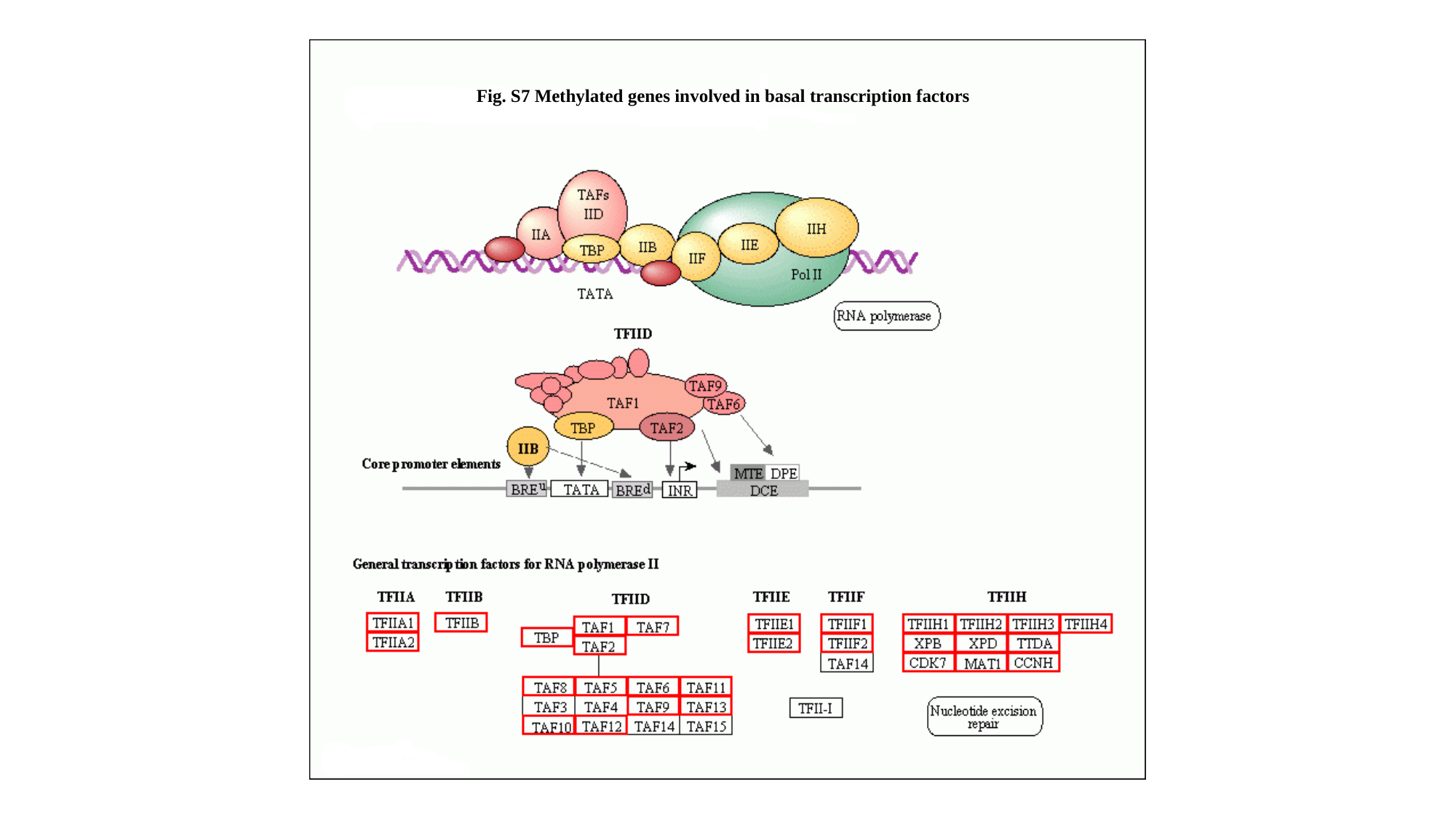

Fig. S7 Methylated genes involved in basal transcription factors

## Slide 8
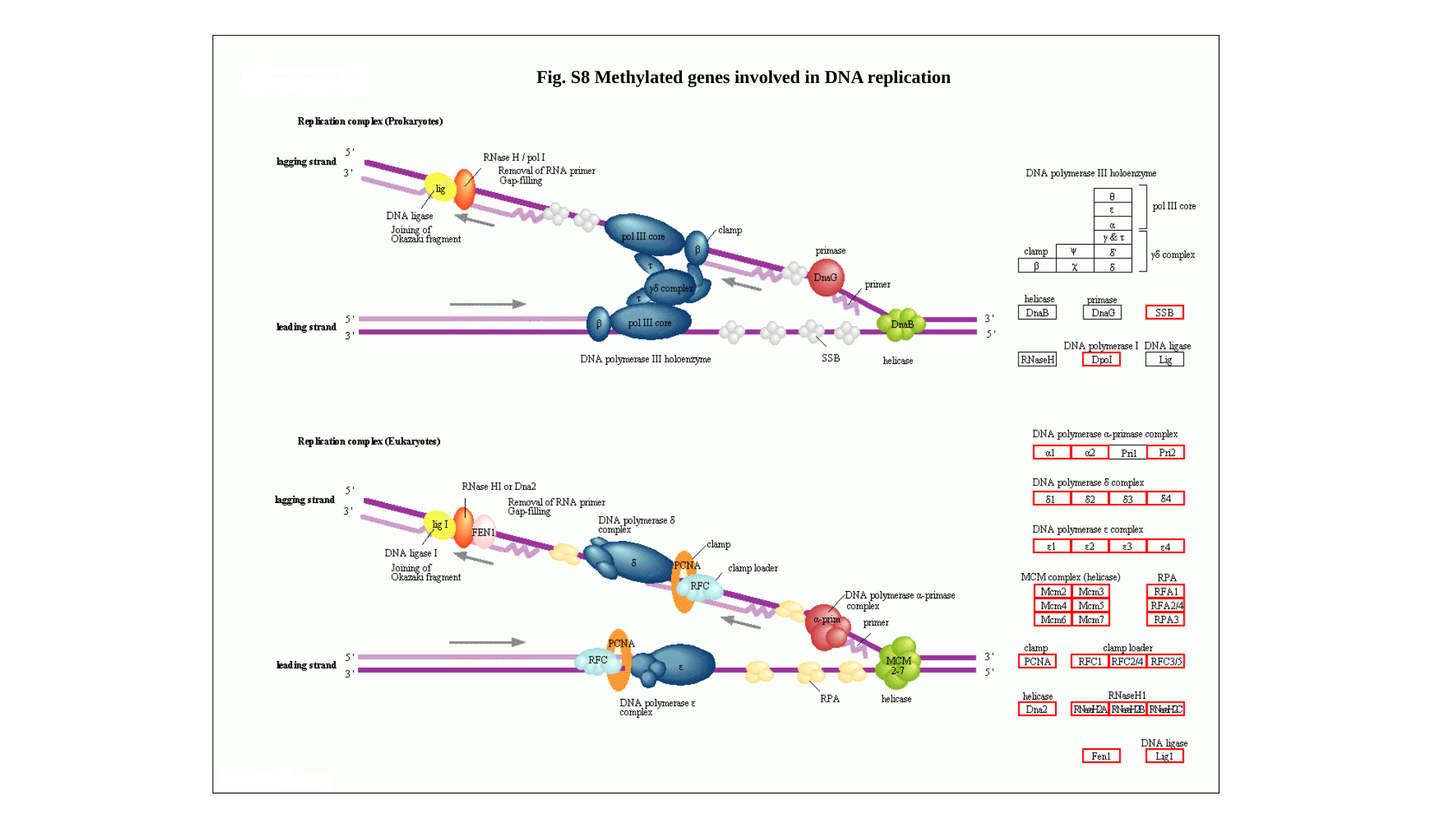

Fig. S8 Methylated genes involved in DNA replication

## Slide 9
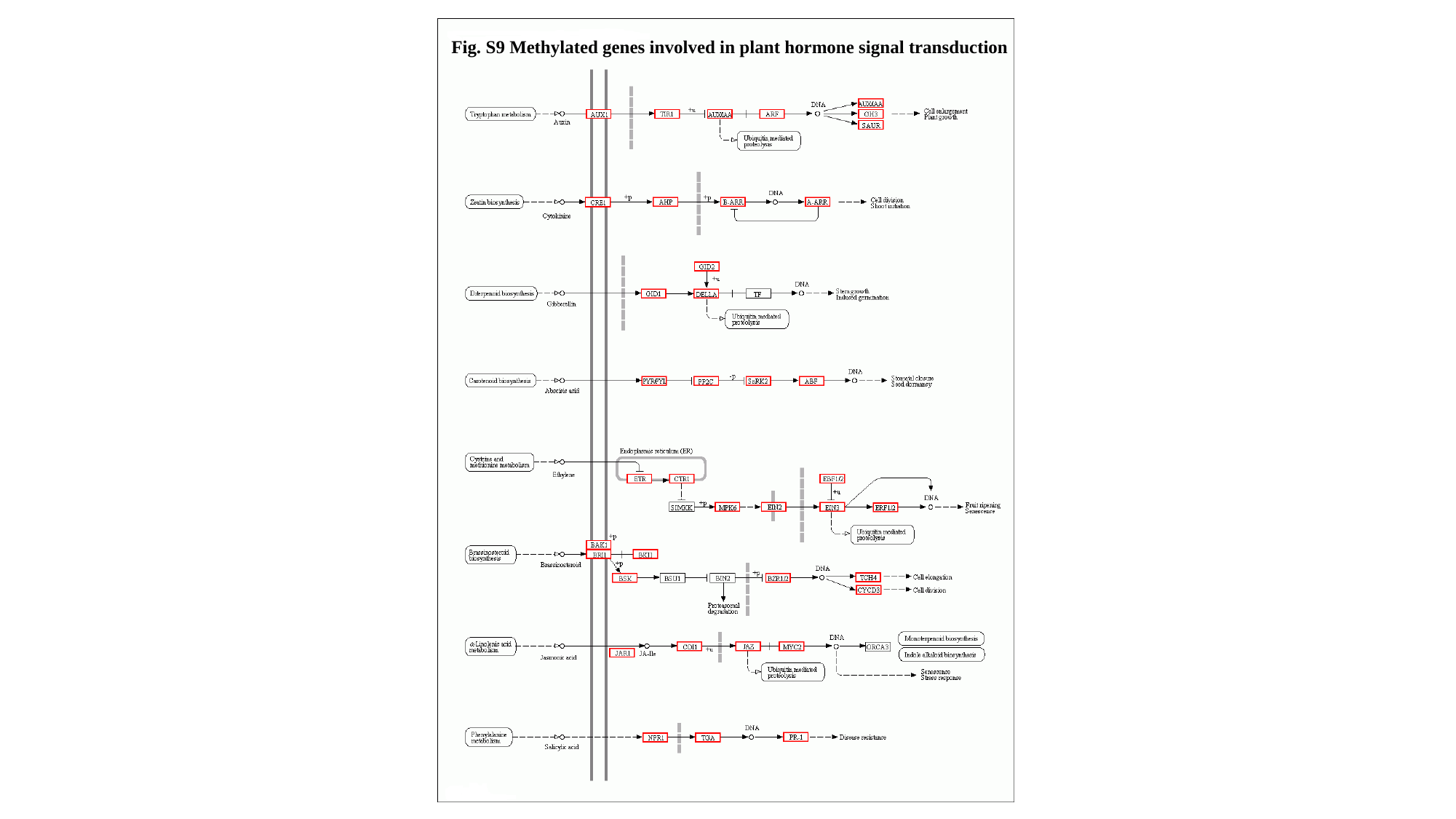

Fig. S9 Methylated genes involved in plant hormone signal transduction
